# Influenza‐like illness in Australia: A comparison of general practice surveillance system with electronic medical records

**DOI:** 10.1111/irv.12774

**Published:** 2020-06-24

**Authors:** Carla De Oliveira Bernardo, David Alejandro González‐Chica, Monique Chilver, Nigel Stocks

**Affiliations:** ^1^ Discipline of General Practice Adelaide Medical School The University of Adelaide Adelaide SA Australia; ^2^ Adelaide Rural Clinical School The University of Adelaide Adelaide SA Australia; ^3^ Australian Partnership for Preparedness Research on Infectious Disease Emergencies (APPRISE) Centre of Research Excellence NHMRC Adelaide SA Australia

**Keywords:** electronic health records, general practice, influenza, respiratory tract infections, syndromic surveillance

## Abstract

Surveillance systems are fundamental to detect infectious disease outbreaks and guide public health responses. We compared influenza‐like illness (ILI) rates for 2015‐2017 using data from the Australian Sentinel Practice Research Network (ASPREN) and electronic medical records from 550 general practices across Australia (MedicineInsight). There was a high correlation between both sources (*r *= .84‐.95) and a consistent higher ILI rate in 2017. Both sources also showed higher ILI rates among women and patients aged 20‐49 years. The use of routinely collected electronic medical records like those in MedicineInsight could be used to complement active influenza surveillance systems in Australia.

## 1 INTRODUCTION

Influenza is an acute respiratory infection that usually causes mild to moderate symptoms, but in some cases can be life‐threatening. The most recent global estimate of influenza‐associated mortality exceeds 645 000 annual deaths.^1^ Current national influenza surveillance systems in Australia consist of general practice and hospital sentinel systems, laboratory‐confirmed notifications and a community based online self‐reported data system.^2^

Influenza‐like illness (ILI), defined as a combination of fever, cough and fatigue,^3^, ^4^ is the recommended indicator for influenza activity surveillance worldwide, along with laboratory testing of a systematic sample.^5^ The Australian Sentinel Practices Research Network (ASPREN, https://aspren.dmac.adelaide.edu.au/) is a surveillance system that actively collects ILI notifications from general practices across Australia.^6^ ASPREN has the advantage of reporting increased ILI activity before a rise in laboratory‐confirmed influenza cases is identified by the National Notifiable Diseases Surveillance System.^6^ Whilst ASPREN data have been the bedrock of general practice influenza surveillance for years,^2^ it has been suggested that active influenza monitoring can be complemented by using routinely collected electronic medical records (EMR) held in general practice databases that also include comprehensive clinical information. Therefore, this study aimed to compare weekly ILI rates and ILI distribution according to sociodemographic characteristics between ASPREN and MedicineInsight, an extensive database with EMR from over 650 general practices from all Australian regions.^7^

## 2 METHODS

### 2.1 Data source

The study included data from ASPREN and MedicineInsight. ASPREN collects notifications of ILI^3^ from a representative sample of Australian practices (one practice per 200 000 individuals in urban and one practice per 50 000‐100 000 individuals in rural areas, according to representation models for sentinel systems).^6^ ASPREN currently collects de‐identified data from more than 200 practices. Most data are electronically collected weekly, via automated extraction or notifications reported in a web‐based system.

MedicineInsight is a national general practice database managed by NPS MedicineWise. De‐identified EMR are extracted monthly from Australian practices located in all jurisdictions, varying by size and type of services offered. Extracted EMR contain sociodemographic and clinical data, laboratory results and prescribed medications. Details of the data collection have been published elsewhere.^7^ MedicineInsight has been used to investigate chronic conditions,^7^ but also patterns of ILI management^4^ and influenza immunization.^8^

### 2.2 Sample selection and data extraction

For this study, ASPREN provided data from 434 GPs in 199 general practices (N = 2 878 458 consultations) between 2015 and 2017. Data included ILI diagnosis (ie GPs must select ILI under the reason for encounter, considering as case definition the combination of fever, cough and fatigue), total weekly consultations, patient's age and sex, and rurality and state/territory of the practice.

MedicineInsight included data from 550 general practices and 4 228 149 patients who had at least one consultation between January/2015 and November/2017 (N = 32 254 306 consultations). A previously developed data extraction algorithm was used to identify all patients with a diagnosis of influenza, ILI diagnosis or ILI symptoms (fever + cough+fatigue).^3^, ^4^ GPs prescriptions of anti‐influenza medication (ie oseltamivir, zanamivir and peramivir) were also coded as positives for ILI even without a recorded ILI diagnosis (7% of all ILI cases), as it commonly happens within primary care data.^4^ All ILI consultations by the same patient within 14 days of the first ILI diagnosis were considered as part of the same event. The diagnosis of other acute respiratory infections [ie upper respiratory tract infections (URTI), acute bronchitis and lower respiratory tract infections (LRTI)] was also extracted from MedicineInsight, as they could influence the recording of ILI because of the similarity in symptoms and known variation in labelling of respiratory illnesses by GPs.^9^ Patient (sex, age, Indigenous status) and practice (rurality and state/territory) characteristics were also extracted from the database.

### 2.3 Data analysis

Weekly, ILI consultation rates (or “attack” consultation rates) were calculated using the number of ILI cases per 1000 consultations. The non‐parametric Spearman's rank correlation was performed to assess the statistical concordance between ASPREN and MedicineInsight. The distribution of ILI according to sociodemographic characteristics was calculated as a percentage among all recorded cases in each data set. Analyses were performed using Stata 16.0. The independent MedicineInsight Data Governance Committee approved the study (protocol 2017‐007) and the Human Research Ethics Committee of the University of Adelaide exempted the study of an ethical review as it used non‐identifiable data.

## 3 RESULTS

**Figure 1:**
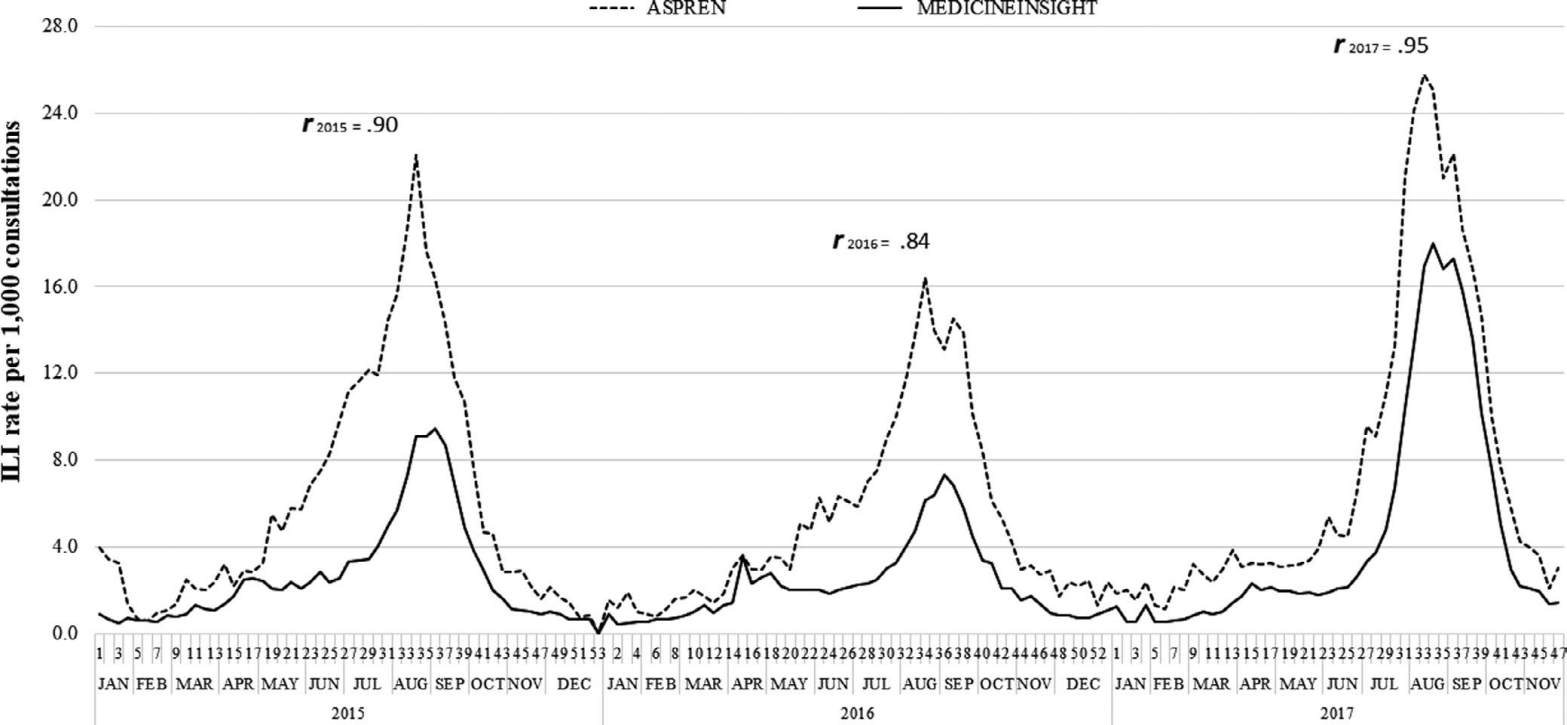
ILI rates per 1000 consultations according to MedicineInsight and ASPREN data sets. Australia, 2015‐2017

The peak of ILI cases in any year was observed between weeks 33 and 36 in both sources (Figure 1). Data from MedicineInsight showed that ILI consultation rates almost doubled in 2017 compared with 2015, reaching a peak of 18.0 per 1000 consultations against 9.4 in 2015. ASPREN results also showed higher ILI rates in 2017, but the difference with 2015 was less pronounced (25.8 vs. 22.1 per 1000 consultations, respectively). Despite this difference, there was a strong positive correlation between both sources (*r *= .84 to .95). Figure 1 also shows ASPREN identified an earlier increase in ILI rates (May‐June) compared with MedicineInsight (July).

**Figure 2:**
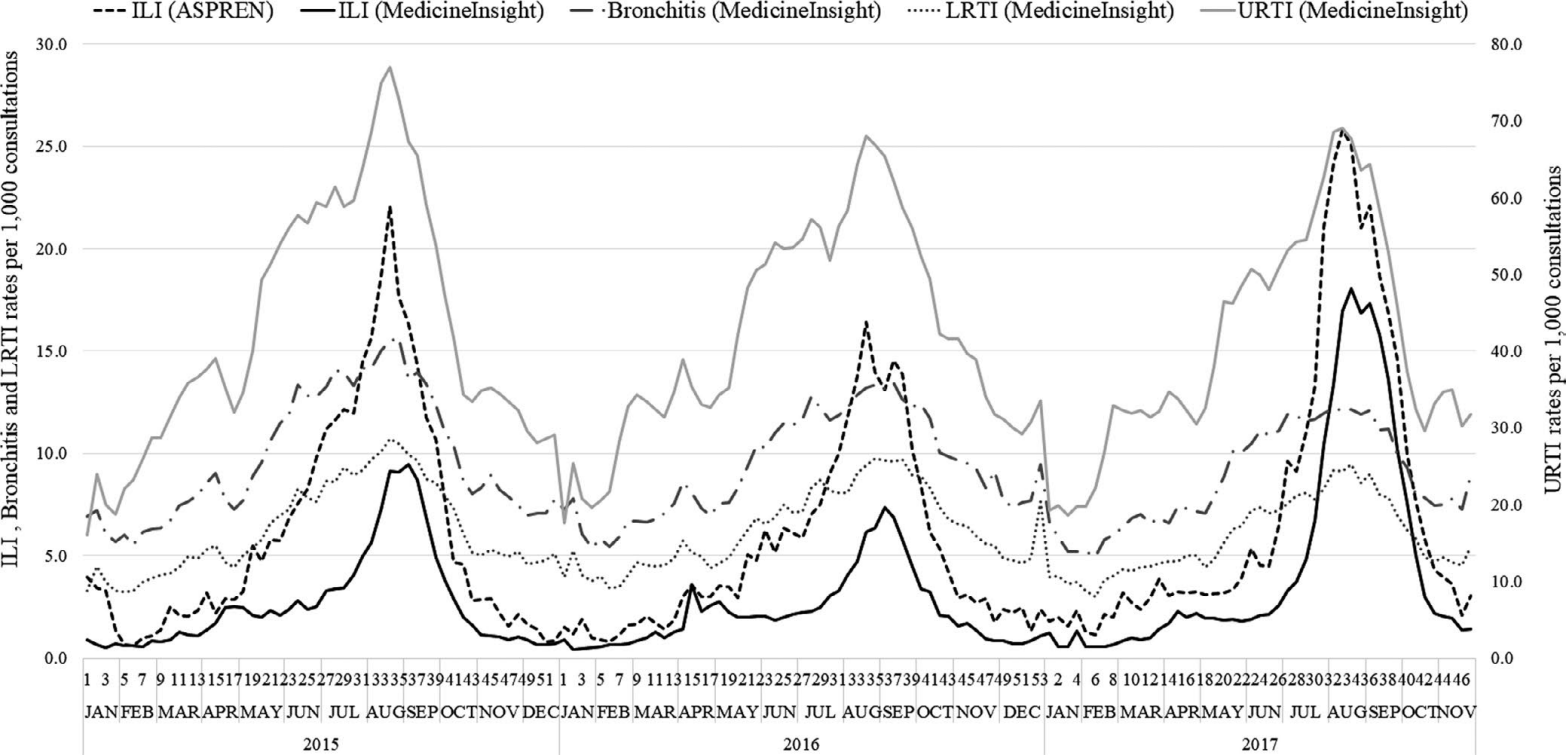
ILI (ASPREN and MedicineInsight), Acute bronchitis, Lower Respiratory Tract Infection (LRTI) and Upper Respiratory Tract Infection (URTI) (MedicineInsight only) rates per 1000 consultations from 2015 to 2017

As shown in Figure 2, the early increase in ASPREN ILI cases coincides with the rise of other acute respiratory infections in MedicineInsight, especially URTI. Table 1 shows a higher proportion of ILI among women or patients aged 20‐49 years in any year in ASPREN and MedicineInsight. New South Wales was the state with the higher proportion of cases in both sources, whilst the frequency of ILI cases in South Australia was less frequent in MedicineInsight.

**Table 1:** Distribution of ILI cases according to patient characteristics. Australia, 2015‐2017

Variable	2015		2016		2017	
	MedicineInsight	ASPREN	MedicineInsight	ASPREN	MedicineInsight	ASPREN
	%	%	%	%	%	%
Sex						
Male	46.4	45.2	46.6	44.6	43.6	44.4
Female	53.6	54.8	53.4	55.4	56.4	55.6
Age						
<5	4.6	11.1	4.5	9.3	5.0	9.5
5‐19	16.1	23.4	12.1	19.3	15.5	20.2
20‐49	42.2	38.3	40.9	39.4	33.0	36.6
50‐64	24.4	16.5	26.2	18.6	28.2	19.7
≥65	12.7	10.7	16.3	13.4	18.4	14.0
Aboriginal/Torres Strait Islander						
No	73.4	78.4	75.2	84.3	76.9	91.9
Yes	1.8	1.4	1.9	5.2	1.9	2.8
Not stated	24.8	20.2	22.9	10.5	21.2	5.3
State						
New South Wales	33.0	34.0	37.6	36.9	44.0	42.1
Victoria	21.3	6.3	18.1	3.5	20.6	4.4
Queensland	21.9	21.3	18.1	17.1	18.5	18.8
Western Australia	10.2	14.5	14.5	24.1	5.6	12.0
Tasmania	6.6	4.2	6.4	4.6	6.1	4.2
South Australia	3.9	16.7	3.2	7.7	2.9	14.0
Northern Territory	1.1	0.9	0.8	2.2	1.8	1.4
Australian Capital Territory	2.1	2.2	1.3	3.9	0.6	3.2
Rurality						
Major cities	67.0	69.8	68.3	68.4	71.9	67.2
Inner regional	20.0	13.8	19.8	15.3	20.0	19.0
Outer regional/remote	13.0	16.3	12.0	16.3	8.1	13.8
Total number of cases	31 813	6388	28 013	4736	38 308	7241

## 4 DISCUSSION

This study aimed to compare weekly ILI rates between ASPREN, a sentinel general practice surveillance system, and MedicineInsight, an extensive EMR general practice database, to identify whether the latter could be used to complement influenza surveillance in Australia. Results showed a high correlation between the two, and consistency regarding the shape of the curves and peaks. The higher rates in 2017 compared with previous years reflects a longer duration and more intense season that year, which was also identified by other surveillance systems in Australia.^10^ Studies in the Unites States and Portugal also found good agreement between sentinel GP surveillance data and alternative databases using EMR, with correlations ranging between 0.78 and 0.99.^11^, ^12^

In ASPREN, a rise in ILI cases started earlier each year compared with MedicineInsight which is probably related to the increase in other acute respiratory infections, particularly URTI, as identified in MedicineInsight. Because GPs can label the same set of respiratory symptoms differently,^9^ it is plausible that ASPREN GPs might code other respiratory infections as ILI because of their role in the sentinel system (ie observer bias). Future studies could address this issue, using regression modelling that takes into consideration the co‐circulation of other pathogens with similar symptoms to influenza during analysis.^13^

The higher incidence of ILI among women in both sources could, in part, be explained by the fact that women attend general practice in Australia more frequently than men.^4^ Alternatively, women may have different social behaviours than men which could increase transmission and therefore infection rates. A prospective cohort in community‐dwelling Australian adults nested within an influenza vaccine effectiveness trial found that women had higher risks of transmission of viral pathogens (eg influenza) after adjustment for living with children.^14^

The lower ILI rates in South Australia in MedicineInsight and Victoria in ASPREN were expected, as the number of practices in these states is underrepresented in the respective databases. However, when the focus is on urban or rural areas, practices from both sources are equally distributed according to remoteness of location and resemble Australian population data (ie 29% of Australians live in rural areas).

In conclusion, both the ASPREN and MedicineInsight provided consistent information on ILI weekly rates and a similar distribution of ILI cases according to sociodemographic characteristics. As a surveillance system, ASPREN also collects respiratory swabs from a systematic sample of patients (~38% of all ILI patients in 2017) that delivers information on the types of influenza viruses circulating, assisting the detection of new strains and providing data for the calculation of vaccine effectiveness.^6^ This additional activity increases the cost of running ASPREN but provides a valuable addition to surveillance data. On the other hand, MedicineInsight has the advantage of providing more comprehensive data that can help in the identification of additional clinical risk factors, including chronic conditions, medications prescribed, vaccination status and the possibility of creating a cohort that can be followed over time. However, the quality of recording in MedicineInsight may vary because information is collected for clinical not research purposes. Laboratory results for influenza are currently not available due to technical issues, although there is the possibility of updating the extracting tool (GRHANITE™) to capture that information. Moreover, the programme currently funded by the Federal Department of Health was established to improve the quality use of medicines not surveillance purposes.^7^ Therefore, to provide routine reports for ILI, as a complementary surveillance system, would require additional funding and a partnership between NPS Medicine Wise, government and researchers. Notwithstanding these barriers, a combination of MedicineInsight and ASPREN and the use of innovative methodological approaches could provide more reliable syndromic and virological information, leading to improved influenza surveillance in Australia.^13^, ^15^

## CONFLICT OF INTEREST

No conflict of interest.

## AUTHOR CONTRIBUTION

**Carla De Oliveira Bernardo:** Conceptualization (equal); Data curation (lead); Formal analysis (lead); Investigation (lead); Methodology (lead); Writing‐original draft (lead); Writing‐review & editing (equal). **David Alejandro González‐Chica:** Conceptualization (equal); Formal analysis (supporting); Investigation (supporting); Methodology (supporting); Writing‐review & editing (equal). **Monique Chilver:** Data curation (supporting); Writing‐review & editing (equal). **Nigel Stocks:** Conceptualization (equal); Investigation (supporting); Project administration (lead); Supervision (lead); Writing‐review & editing (equal). 
